# Severe Lower Extremity Cellulitis Caused by an Unusual Pathogen: Haemophilus Influenzae Type F

**DOI:** 10.7759/cureus.7819

**Published:** 2020-04-24

**Authors:** Victoria Bengualid, Juanita Rufran, Marcin Romancyzk, Russell F Trahan, Judith Berger

**Affiliations:** 1 Infectious Diseases, St. Barnabas Hospital Health System, Bronx, USA; 2 Podiatry, St. Barnabas Hospital Health System, Bronx, USA; 3 Surgery, St. Barnabas Hospital Health System, Bronx, USA

**Keywords:** cellulitis, haemophilus influenza, type f

## Abstract

We present a case of unusual cellulitis of the lower extremities caused by *Haemophilus influenzae *(HI). A 64-year-old female with human immunodeficiency virus (HIV) with a suppressed viral load on treatment, presented with severe, very painful cellulitis of her lower extremity. CT scan did not show any gas or collections; however, she was taken to the operating room for concern of necrotizing fasciitis but no evidence of deep tissue involvement was found. Blood culture and wound culture were positive forHI type F (HiF), a newly emergent pathogenic capsulatedHI that has emerged post-HI type B (HiB) vaccination.

## Introduction

Cellulitis, a skin infection causing redness, warmth, and edema, is most often caused by the gram-positive organisms Streptococcus and Staphylococcus. We present a case of gram-negative cellulitis caused by *Haemophilus influenzae* (HI), an organism known more for its propensity to cause disease in children, manifested by respiratory infections and meningitis, than lower extremity cellulitis. We present an adult patient with severe foot cellulitis caused by HI type F (HiF), an emerging subtype post-HI type B (HiB) vaccine.

## Case presentation

A 64-year-old female was a history of human immunodeficiency virus (HIV) (on antiretroviral treatment with a cluster of differentiation antigen 4 (CD4) 530/ul and a viral load of 40 copies/ml) and asthma, was admitted for severe pain and redness of her right foot.

One week prior to admission, the patient felt pain in her foot. She also noted a dry cough with minimal sputum production. She went to another hospital as the pain became severe. There, she was treated for cellulitis with intravenous antibiotics for one day. She was discharged on oral antibiotics but did not fill her prescription. The patient developed fever at home, with increased swelling and pain of her foot. She presented to our emergency room one week after her prior discharge from the hospital. She denied any trauma to her foot, drug use, recent travel, or exposure to animals. 

**Figure 1:**
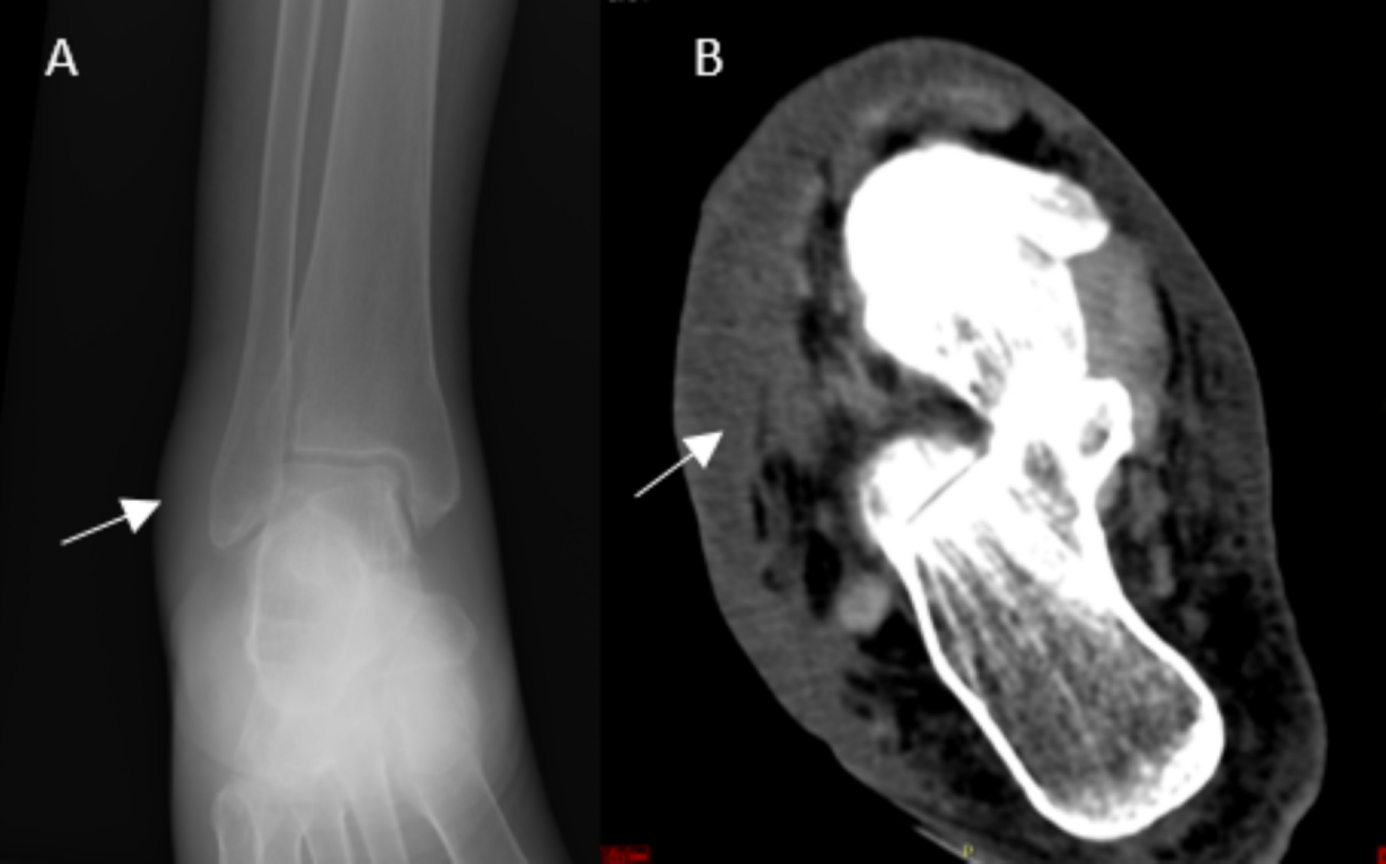
Imaging of the right foot A: X-ray of the right foot. Soft tissue swelling (arrow) around the lateral malleolus. No gas or bony erosions noted. B: CT scan of the right foot. Soft tissue swelling with subcutaneous edema and fluid (arrow) with skin thickening.

**Figure 2:**
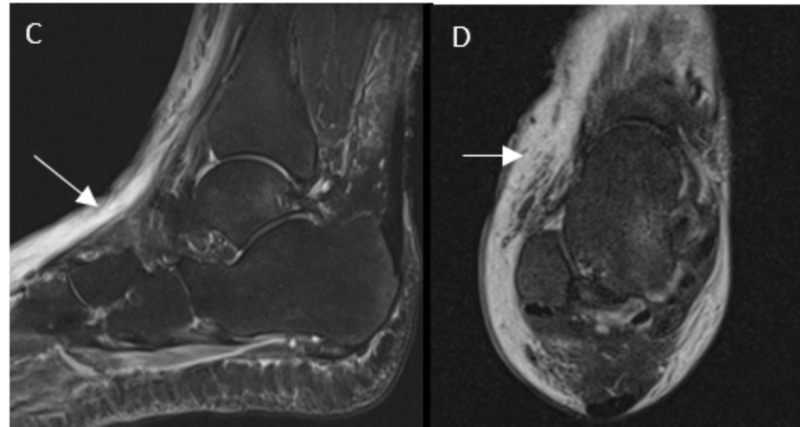
MRI scan of the right foot C: Sagittal and D: axial images showing dorsal ankle and soft tissue swelling with edema (arrow).

On physical exam, her temperature was 101.6° F. She had a dry cough but no sinus congestion or pharyngitis. Physical exam was remarkable for clear lungs and erythematous swollen right foot with severe pain. Dorsalis pedis pulse could not be felt secondary to the swelling. The patient was started on vancomycin and cefepime. The patient had imaging of her foot; X-ray was notable for swelling without any evidence of periosteal elevation, and a CT scan was consistent with cellulitis without gas or collections (Figure 1). An MRI scan was also consistent with cellulitis and tissue edema without bone involvement (Figure 2). 

Chest X-ray and sinus X-rays were negative. On hospital day two, the patient was still febrile but felt less pain. She developed a bulla with clear fluid at the site of cellulitis. Her white blood cell (WBC) count was 21 10^3^/ul. 

On hospital day three, her cough had resolved. Her foot was still edematous, and painful. A small fluid collection was palpated at the site of the cellulitis. Because of concern for necrotizing fasciitis, she was taken to the operating room for debridement. The collection was drained and the tissue was debrided. 

Admission blood cultures, as well as the fluid culture taken in the operating room, grew HiF and was beta-lactamase positive. Her antibiotics were switched to piperacillin and tazobactam. Her foot continued to improve and she became afebrile on hospital day six. Her WBC count decreased to 9.6 10^3^/ul. Her antibiotics were switched to oral ciprofloxacin and metronidazole. 

On hospital day 13, she was taken to the operating room for wound irrigation and placement of a wound vac. She was discharged on hospital day 16 to complete three weeks of treatment. The patient was followed in a wound clinic where she received hyperbaric oxygen and wound management. She returned to the operating room one month after discharge for bone biopsy of her first, second, and fourth digit because of concern of possible osteomyelitis. The pathology was negative for osteomyelitis. The wound continued to heal with conservative wound management. 

## Discussion

HI is a gram-negative bacteria that normally colonize the respiratory tract and is known to cause pneumonia, meningitis, periorbital cellulitis, and epiglottitis. HI cellulitis is rare and mainly described in children. The most common sites of HI cellulitis are the head, neck, and chest as HI is known to colonize the respiratory tract and the oral cavity.

Cellulitis of the extremities secondary to HI is even more uncommon. A review of the literature yielded eight cases of cellulitis of the extremities in adults and another four cases in children [1-11]. All these patients presented with HI bacteremia.

The ages in the eight adult cases ranged from 42 to 86 years. Four patients had a form of immunosuppression (steroid use, chronic lymphocytic leukemia, immunoglobulin G (IgG) 4 deficiency, and HIV with a CD4 count of 530/ul (as in our case) [1,5,8]. One case involved solely the upper extremities and a second involved the hand and foot [2,6]. Cellulitis occurred in one lower extremity in the remaining cases.

Our case was notable for the severity of the cellulitis. Our patient presented with intense pain and bullae (one other case also presented with bullae) raising the concern for necrotizing fasciitis [6]. Our patient was taken to the operating room for debridement but on inspection, the deeper layers were not involved. The presentation of severe cellulitis with concerns for necrotizing fasciitis was also present in two of the case reports [6-7]. 

It remains unclear from these case reports if HI causes localized cellulitis which then seeds the blood or if the blood infection preceded the cellulitis. Two patients had a potential history of skin trauma; our case and a second case had concomitant upper respiratory tract symptoms, while the remaining five cases had no history of trauma, respiratory or dental symptoms [6-8]. 

HI is divided into typeable strains (those strains that have a capsule) and non-typeable strains (those without a capsule). In these nine adult cases, four cases occurred in the pre-HIB vaccine era; three cases were infected with HiB and one infection was caused by HI non-B [1-4]. The remaining five cases occurred after the introduction of hepatitis HiB vaccine. Treatment ranged from seven days to six weeks.

In the wake of the dramatic decrease in HiB infection post-HiB conjugate vaccination, non-typeable and some capsulated strain have emerged as pathogens. A study done by Rubach et al. in Utah, noted that in the 20 years post introduction of the HiB vaccine in Utah, the incidence of invasive HI has shifted toward an older population (51% of the cases were in adults 65 years or older) [12]. Non-typeable HI was associated with the highest incidence of invasive disease followed by the capsulated type F.

The same pattern described in Utah of the emergence of non-typeable HI, encapsulated type F, and invasive disease affecting an older population has been described in Sweden, England, Wales, Spain, and Japan [13-17]. Another encapsulated type, strain A, has emerged as a pathogen in children in Utah, Alaska and the Navajo Nation [12]. In the five case reports of HI causing cellulitis in adults in the post-vaccine era (four case reports and our case), four were due to type F confirming the trends that have been described globally.

## Conclusions

HI cellulitis in adults of the lower extremity is extremely rare with only eight other cases described in the literature. In the post-vaccination era, cellulitis of the extremities is mainly due to the emergence of the more pathogenic encapsulated subtype F. Presentation of HI cellulitis of the extremities has been described as severe and can be confused with necrotizing fasciitis. However surgical exploration does not show involvement of the fascia or the subcutaneous tissues. All the cases were accompanied by bacteremia; hence, stressing the importance of blood culture in making the diagnosis.
